# Effects of hemoperfusion and continuous renal replacement therapy on patient survival following paraquat poisoning

**DOI:** 10.1371/journal.pone.0181207

**Published:** 2017-07-13

**Authors:** Yadong Wang, Yao Chen, Lu Mao, Guangju Zhao, Guangliang Hong, Mengfang Li, Bin Wu, Xiaorong Chen, Meng Tan, Na Wang, Zhongqiu Lu

**Affiliations:** Department of Emergency, The First Affiliated Hospital of Wenzhou Medical University, Wenzhou, Zhejiang, China; Fudan University, CHINA

## Abstract

Mortality in patients with paraquat (PQ) poisoning is related to plasma PQ levels. Concentrations lower than 5,000 ng/mL are considered critical but curable. This study assessed the effects of hemoperfusion (HP) and continuous renal replacement therapy (CRRT) on the survival of PQ-poisoned patients with plasma PQ levels below 5,000ng/mL. We analyzed the records of 164 patients with PQ poisoning who were treated at the First Affiliated Hospital of Wenzhou Medical University in China between January 2011 and May 2015. We divided these patients into six sub-groups based on baseline plasma PQ levels and treatment, compared their clinical characteristics, and analyzed their survival rates. Patient sub-groups did not differ in terms of age, sex, time between poisoning and hospital admission, or time to first gavage. Biochemical indicators improved over time in all sub-groups following treatment, and the combined HP and CRRT treatment yielded better results than HP or CRRT alone. Fatality rates in the three treatment sub-groups did not differ among patients with baseline plasma PQ levels of 50–1,000 ng/mL, but in patients with 1,000–5,000 ng/mL levels, the mortality rate was 59.2% (HP treatment group), 48% (CRRT treatment group), and 37.9% (combined treatment group). Mortality rates were higher 10–30 days after hospitalization than in the first 10 days after admission. In the early stages of PQ poisoning, CRRT is effective in reducing patient fatality rates, particularly when combined with HP. Our data could be useful in increasing survival in acute PQ poisoning patients.

## Introduction

Paraquat (PQ) is a nonselective herbicide that has been widely used worldwide since the 1960s. Due to its availability, low toxic dose, and relatively low cost, PQ is used to commit suicide in developing countries [1, 2]. Although safely used in agriculture, PQ intoxication is a serious public health problem, and results in an estimated 60–70% mortality [3, 4]. Because no specific antidote or conclusively effective treatment having been found, an acute ingestion of 7–8 mL PQ can cause serious effects such as liver, lung, kidney, and heart failure, leading directly to death [5, 6].

Various therapeutic techniques are applied to clinical intervention in PQ poisoning. To protect organ function, the routine treatment protocol includes reducing the absorption of PQ in the gastrointestinal tract by gastric lavage, increasing PQ elimination from plasma by hemoperfusion (HP) and hemodialysis (HD), repeated application of anti-inflammatory agents such as methylprednisolone (MP), cyclophosphamide (CTX) pulse immunosuppressive therapy, administration of antioxidants, and maintenance of vital functions [7–10]. Among these techniques, HP has been validated as the most effective method to clear PQ from blood [11, 12]. However, HP may result in complications such as thrombocytopenia, leukopenia, hypoglycemia, and some reduction in clotting factors. Risk of bleeding is higher because of high heparin doses and a reduction in platelets and clotting factors [13]. Continuous renal replacement therapy (CRRT) is most commonly used in an intensive care unit setting, where it is either given as 8- or 12-h treatments, also termed slow extended hemofiltration (SLEF). With slow blood flow rates and access through a central venous catheter placed in one of the large central veins, CRRT can keep blood from clotting even if heparin and regional citrate are used for anti-coagulation [14, 15]. Plasma PQ levels have been shown to be related to liver, kidney, and coagulation functions, and may be used as a clinical index in assessing the prognosis of PQ-poisoned patients [7]. Ingestion of more than 5,000 ng/mL of PQ generally results in 100% mortality, and the lower the plasma concentration, the higher the relative survival rate [7]. Although HP has been validated as an effective treatment, the effects of combined treatment with HP and CRRT on the survival of patients with PQ poisoning at different concentrations, particularly PQ levels from 1,000 to 5,000 ng/mL, is still unclear.

Based on long-time monitoring of the effects of HP and CRRT at different PQ concentrations, we performed a retrospective study on the prognosis and survival of PQ-poisoned patients admitted to the First Afflliated Hospital of Wenzhou Medical University in China. The various clinical indices included the time of treatment, and biochemistry parameters were analyzed to assess the effects of HP and CRRT on survival time.

## Materials and methods

### Ethics statement

This study was approved by the Medical Ethics Committee of The First Afflliated Hospital of Wenzhou Medical University, and conducted in accordance with the Declaration of Helsinki. Because this study was a retrospective investigation of existing data, written informed consent from patients was not required. However, informed consent regarding the treatment risk following acute PQ poisoning was obtained from all patients upon their initial admission. All information gathered from PQ-poisoned patients was anonymized and securely protected. All data were only available to the investigators.

### Patients and groups

The poisoning history of PQ-poisoned patients was provided by the patients themselves or their families. We reviewed the medical records of patients admitted to the First Afflliated Hospital of Wenzhou Medical University in China with acute PQ poisoning between January 1, 2011 and May 31, 2015. During this period, 215 patients with PQ poisoning were admitted to the hospital; 164 patients were eventually included in the study on the basis of inclusion and exclusion criteria. Inclusion criteria were (1) a clear history of PQ poisoning and PQ detected in blood; (2) patient age between 15 and 65 years; (3) hospital admission within 24 h of poisoning without pretreatment; (4) no history of serious chronic disease; (5) no contraindications of HP treatment. Exclusion criteria were (1) PQ poisoning patients who died within 24 h; (2) patients with other pesticide poisoning; (3) pregnant patients; (4) patients with a history of alcoholism; and (5) patients with serum PQ levels above 5000 ng/mL. The initial plasma PQ concentration was measured immediately upon the patient’s arrival in the emergency department of the hospital and prior to receiving any treatment. The PQ-poisoned patients were divided into two different groups (low PQ [LPQ] group and high PQ [HPQ] group) according to their initial plasma PQ concentrations. PQ concentrations in the LPQ group were below 1000 ng/mL. Each group was randomly sub-divided into three sub-groups based on treatment: HP sub-group, CRRT sub-group, and HP+CRRT sub-group. The size of each group was as follows: (1) LPQ-HP, 28 cases; (2) LPQ-CRRT, 26 cases; (3) LPQ-HP-CRRT, 29 cases; (4) HPQ-HP, 27 cases; (5) HPQ-CRRT, 25 cases; (6) HPQ-HP-CRRT, 29 cases. All data obtained from the PQ-poisoned patients were recorded and standardized in a Microsoft Excel spreadsheet by two emergency department physicians who were unaware of the purpose of this investigation. The study’s design and data analyses had been finished by all authors, and the results’s review and the paper had been completed by Yadong Wang, Guangliang Hong and Zhongqiu Lu.

### Treatments

#### Conventional treatments

Gastrolavage with 2% NaHCO_3_ and catharsis with 20% mannitol are the conventional initial treatments [16], and both were used in this study’s cohort. Diuresis and other symptomatic treatments were performed next. Water-electrolyte imbalances in patients were corrected by multiple rehydration sessions. The patients received high-dose intravenous MP (80–320 mg every six hours) combined with CTX (0.2–1 g every day for 3 days) to prevent pulmonary fibrosis as early as possible. High-dose vitamin C and glutathione were administered to scavenge oxygen free radicals. MP and/or CTX therapy was administered repeatedly according to the condition of the patient. Stomach- and liver-protective drugs, such as magnesium isoglycyrrhizinate and polyene phosphatidylcholine, were applied, along with effective antimicrobials to prevent infections. To avoid a partial pressure of oxygen (PaO_2_) <40 mmHg or adult respiratory distress syndrome (ARDS), treatment by oxygen inhalation or ventilator was carried out with positive end-expiratory pressure (PEEP) positive pressure ventilation.

The therapeutic effect on PQ-poisoned patients was assessed via their vital signs, temperature, respiration, heart rate, blood pressure, and blood indices such as arterial blood gas, liver function, and kidney function. A good treatment outcome was considered a body temperature of 37°C, respiration rate below 20 breaths per minute, heart rate within 60–100 beats per minute, blood pressure in normal level (systolic value: 91–119, diastolic value: 61–79), and a marked improvement in blood indices. The survival time was calculated from the time of PQ ingestion to the time the patient was discharged from the hospital.

#### HP and CRRT treatments

HP and/or CRRT therapies were initiated at the same time as gastric lavage to prevent renal failure and reduce plasma PQ levels. These two treatment methods for PQ poisoning are key therapies [16, 17].

HP treatment: The patients were immediately administered HP through femoral venous catheters at a blood flow rate of 200 mL/min. The HP apparatus consisted of polypropylene housing material, cellulose, and an activated charcoal adsorbent [16]. HP was conducted for 3–4 h, once daily, and the courses of HP therapy were determined by plasma PQ concentrations until they dropped below 50 ng/mL, as well as by clinical characteristics.

CRRT treatment: Blood was filtered and returned to the patient with replacement fluid. The volume of replacement fluid was adjusted according to individual patient requirements, and its components were adjusted based on their levels. Blood flow rates were in the range of 100–200 mL/min, and access was achieved through a central venous catheter placed in one of the large central veins. The CRRT was given as an 8–12-h daily treatment.

An ADM-08 dialysis machine (Fresenius, Bad Homburg vor der Höhe, Germany), BM-25 continuous bedside blood purification machine (Baxter, Deerfield, IL, USA), AV-600S polysulfone membrane filter (Fresenius), P2S plasma separator (Fresenius), and 11.5-Fr double lumen hemodialysis catheters (Arrow Teleflex, Wayne, PA, USA) were used for the CRRT.

HP+CRRT treatment: In the combined treatment, HP was applied first, followed by CRRT using the methods above once daily.

### Blood biochemistry

Blood samples were collected into evacuated tubes containing a separation gel and separated by immediate centrifugation at 1500 ×*g* for 10 min at 4°C. The obtained plasma was analyzed with an automatic biochemical analyzer (Drawell 800T/H, Shanghai, China) for liver, kidney, and coagulation function. Based on previous research [7], we selected the biochemical parameters that were significantly correlated with plasma PQ concentration as the evaluation factors. Blood liver parameters included total bilirubin, direct bilirubin, indirect bilirubin, alanine aminotransferase (ALT), aspartate transaminase (AST), and the ratio of ALT and AST (ALT/AST). Blood kidney parameters consisted of blood urea nitrogen and creatinine levels. Coagulation function values included prothrombin time and prothrombin activity. These parameters were analyzed at the same time as PQ plasma concentration when the patients arrived at the hospital.

### Data analysis

The data were analyzed using SPSS (version 19.0, IBM SPSS, Armonk, NY, USA). Results are presented as means ±standard deviation. The level of significance was set at *p*<0.05. Categorical variables were analyzed using the chi-squared test. The variables were analyzed via nonparametric test and the other indices were analyzed via ANOVA. To assess the relationship between treatment protocols and mortality, the multivariate Cox regression survival curves with the Cox proportional hazard model were generated. The primary dependent variable was the survival time, measured in days. The treatment outcomes of PQ-poisoned patients were entered as categorical variables (1: Better; 0: Worse or death). Multivariate adjustment was made separately for age, sex, time from PQ ingestion to arrival at the hospital, time from PQ ingestion to first gastric lavage, time from PQ ingestion to first HP/CRRT/HP+CRRT treatment, and liver, kidney, and coagulation function.

## Results

### Patient characteristics

Records of 164 patients (76 men, 88 women) with a PQ poisoning history met the criteria and were included in this study. The general clinical data of patients in the six sub-groups are shown in Table 1. The baseline biochemical parameters are listed in the baseline column in Table 2. Eighty-three patients with plasma PQ concentrations between 50 ng/mL and 1,000 ng/mL were assigned to three treatment sub-groups. There was no statistically significant difference (*p*>0.05) in sex, age, admission time, time to first gavage, time to first HP/CRRT/HP+CRRT treatment, length of hospital stay, survival, or initial or improved biochemical parameters between the groups. The 81 patients with plasma PQ levels of 1000–5000 ng/mL exhibited comparable biochemical parameters, with the exception of survival or improvement, as calculated by variance analysis. These data indicate that the treatments were comparable across the different sub-groups.

**Table 1:** Clinical characteristics of paraquat (PQ)-poisoned patients (n = 164).

characteristic	50–1,000 ng/mL plasma PQ level			1,000–5,000 ng/mL plasma PQ level		
	HP	CRRT	HP+CRRT	HP	CRRT	HP+CRRT
**Age (years)**	35.61 ±9.32	36.53 ±11.51	34.52 ±8.43	35.41 ±12.33	34.21 ±12.55	36.72 ±13.23
**Male/female**	12/16	11/15	14/15	15/12	12/13	12/17
**Time to hospital admission (h)**	12.53 ±4.42	11.64 ±6.36	13.8 2 ±5.53	7.35 ±3.64	8.52 ±4.28	7.82 ±4.74
**Time to first gavage (h)**	5.52 ±2.54	4.47 ±3.42	4.81 ±3.52	2.56 ±1.34	2.43 ±1.67	2.82 ±1.47
**Time to first HP/CRRT /HP+CRRT(h)**	17.32 ±7.25	16.78 ±8.27	17.64 ±0.152	15.67 ±2.38	15.82 ±1.64	15.72 ±1.45
**Length of hospital stay** **(days)**	17.72 ±2.65	17.67 ±2.38	17.62 ±2.55	15.43 ±4.7	15.73 ±4.44	15.52 ±4.78
**Survival or improvement**	26/28 (92.8%)	24/26 (92.3%)	27/29 (93.1%)	11/27 (40.7%)	13/25 (52%)	18/29 (62.1%)

Continuous variables are presented as means ± standard deviation. Abbreviations: HP, hemoperfusion; CRRT, continuous renal replacement therapy; HP+CRRT, combined treatment.

**Table 2:** Blood biochemical parameters of patients with low plasma paraquat (PQ) levels over time (n = 83).

Parameter	50 ng/mL < PQ level < 1,000 ng/mL								
	HP			CRRT			HP+CRRT		
	Baseline	Day 5	Day 10	Baseline	Day 5	Day 10	Baseline	Day 5	Day 10
**Total bilirubin** **(μmol/L)**	26.53 ±33.82	36.76 ±22.61	46.41 ±21.54	27.59 ±34.86	33.66 ±23.54	42.41 ±22.72	26.62 ±33.35	30.12 ±24.42	39.23 ±23.27
**Direct bilirubin** **(μmol/L)**	16.12 ±33.2	24.21 ±23.32	30.32 ±22.43	17.32 ±31.21	25.53 ±21.19	29.45 ±22.72	16.67 ±31.21	24.43 ±22.50	29.78 ±23.63
**Indirect bilirubin** **(μmol/L)**	12.34 ±6.32	12.34 ±5.53	12.64 ±5.61	11.53 ±5.65	11.57 ±6.52	11.72 ±6.75	11.53 ±6.31	11.45 ±6.54	11.57 ±6.94
**Alanine aminotransferase** **(U/L)**	79.75 ±110.23	123.67 ±123.34	154.55 ±124.34	78.74 ±107.42	124.42±112.4	159.72 ±119.47	79.63 ±111.32	133.57 ±87.35	162.66 ±79.68
**Aspartate transaminase** **(U/L)**	70.54 ±78.64	92.67 ±67.56	118.67 ±73.35	69.51 ±79.36	89.67 ±78.42	121.56 ±75.34	69.51 ±80.12	88.53 ±76.31	120.45 ±77.48
**Blood urea nitrogen** **(U/L)**	6.86 ±6.45	7.43 ±6.45	7.37 ±6.53	7.25 ±6.35	7.54 ±6.45	7.32 ±6.52	7.12 ±7.83	7.61 ±6.38	7.47 ±6.57
**Creatinine** **(U/L)**	129.13 ±137.89	156.35 ±110.5	174.24 ±120.18	130.34 ±138.58	158.42±122.3	171.36 ±120.47	130.18 ±139.52	155.67 ±130.49	169.41 ±125.53
**Prothrombin time(s)**	14.65 ±5.27	15.32 ±5.23	14.87 ±4.89	13.98 ±5.17	14.79 ±5.54	15.36 ±5.57	14.72 ±5.21	15.46 ±4.34	14.69 ±4.65
**Prothrombin activity(%)**	82.13 ±13.12	81.78 ±11.42	82.33 ±12.23	81.85 ±13.40	81.56 ±13.52	82.58 ±12.84	81.76 ±13.52	81.65 ±12.45	82.16 ±12.62
**Partial pressure** **of oxygen** **(mmHg)**	92.35 ±11.62	91.23 ±10.86	92.43 ±11.54	91.79 ±12.13	91.42 ±12.37	92.35 ±11.85	93.13 ±11.34	92.34 ±12.43	92.57 ±12.17
**Creatine kinase myocardial B fraction** **(U/L)**	14.32 ±8.34	16.76 ±7.67	16.38 ±7.37	14.11 ±8.24	16.33 ±8.26	16.62 ±9.13	14.31 ±8.26	16.35 ±8.42	16.24 ±8.23

Continuous variables are presented as means ± standard deviation. Abbreviations: HP, hemoperfusion; CRRT, continuous renal replacement therapy; HP+CRRT, combined treatment.

### Patient clinical parameters during treatment

Table 2 shows the biochemical parameters of patients with plasma PQ concentrations ranging from 50 to 1,000 ng/mL. At admission (baseline), these parameters did not differ among the three treatment sub-groups (HP, CRRT, and HP+CRRT). With the progression of toxicity, multi-organ damage became a serious concern. Levels of total bilirubin, direct bilirubin, alanine aminotransferase, aspartate transaminase, and creatinine rose in every sub-group over time, and were highest on day 7. In contrast, levels of indirect bilirubin, blood urea nitrogen, prothrombin time, prothrombin activity, and partial pressure of oxygen did not change between admission and day 10 in any of the three treatment sub-groups. These data revealed that low plasma PQ concentration mainly damaged patient’s liver and renal function, indicating more attention to the liver and kidney function protection.

In patients with plasma PQ levels ranging from 1,000 to 5,000 ng/mL, we analyzed the effect of different treatments (HP, CRRT, and HP+CRRT) at three time points (baseline, day 5, and day 10). As shown in Table 3, no significant differences in biochemical parameters were observed at baseline among the three tested sub-groups. However, compared with baseline, all parameters indicated severe multi-organ damage on day 5 and day 10 in each sub-group, although indirect bilirubin values were unchanged. Both CRRT and HP+CRRT therapies were superior to HP in improving patients’ vital signs, and the HP+CRRT combination treatment was more effective than CRRT. Analysis showed that the three treatments resulted in markedly different effects in patients with plasma PQ concentrations between 1,000 and 5,000 ng/mL.

**Table 3:** Blood biochemical parameters of patients with high plasma paraquat (PQ) levels over time (n = 81).

Parameter	1,000 ng/mL < PQ level < 5,000 ng/mL								
	HP			CRRT			HP+CRRT		
	Baseline	Day 5	Day 10	Baseline	Day 5	Day 10	Baseline	Day 5	Day 10
**Total bilirubin** **(μmol/L)**	25.59 ±32.35	47.53 ±23.63	58.65 ±24.56	26. 62 ±34.22	36.58 ±24.55	46.38 ±23.48	25. 53 ±33.82	31.43 ±22.34	39.45 ±22.63
**Direct bilirubin** **(μmol/L)**	15.65 ±32.5	28.21 ±22.41	37.41 ±23.32	16.23 ±32.15	22.31 ±20.12	30.34 ±21.58	15.67 ±32.34	18.68 ±20.52	25.65 ±22.35
**Indirect bilirubin** **(μmol/L)**	12.64 ±5.47	12.53 ±5.41	12.88 ±5.35	12.34 ±5.56	12.75 ±6.32	12.72 ±5.43	12.36 ±6.12	12.55 ±6.29	12.65 ±6.47
**Alanine aminotransferase** **(U/L)**	81.74 ±112.34	176.52 ±126.41	267.31 ±131.46	79.85 ±117.52	153.65±89.43	236.74 ±102.45	79.34 ±113.76	127.68 ±85.53	184.63 ±78.46
**Aspartate transaminase** **(U/L)**	71.46 ±73.41	192.43 ±71.35	248.63 ±75.58	69.23 ±74.42	165.52±67.32	211.43 ±62.38	70.54 ±74.26	135.42 ±63.33	163.51 ±65.45
**Blood urea nitrogen (U/L)**	7.53 ±6.62	15.58 ±7.46	23.52 ±6.59	7.43 ±7.12	12.42 ±6.45	17.47 ±6.62	7.31 ±7.11	9.23 ±5.36	13.56 ±5.17
**Creatinine** **(U/L)**	124.31 ±134.34	256.35 ±123.6	374.24 ±133.26	127.34 ±135.57	223.41±117.5	333.41 ±116.52	127.43 ±134.17	186.46 ±112.47	289.56 ±118.46
**Prothrombin time(s)**	13.42 ±4.78	15.41 ±5.13	17.63 ±5.23	13.64 ±5.22	15.57 ±5.41	17.47 ±5.45	13.39 ±5.27	14.58 ±4.52	16.79 ±4.45
**Prothrombin activity (%)**	81.48 ±12.24	75.57 ±12.25	71.15 ±12.41	82.36 ±13.14	74.36 ±12.62	70.65 ±12.44	81.41 ±12.71	73.56 ±11.42	70.12 ±11.49
**Partial pressure of oxygen(mmHg)**	91.58 ±11.32	87.15 ±10.46	82.42 ±10.41	91.62 ±11.43	86.43 ±12.13	90.68 ±10.47	92.14 ±11.42	88.58 ±11.55	91.64 ±11.32
**Creatine kinase myocardial B fraction(U/L)**	13.46 ±8.23	15.52 ±8.64	18.57 ±7.41	13.53 ±8.67	16.42 ±7.67	17.68 ±8.32	13.64 ±8.27	15.66 ±7.32	16.48 ±6.53

Continuous variables are presented as means ± standard deviation. Abbreviations: HP, hemoperfusion; CRRT, continuous renal replacement therapy; HP+CRRT, combined treatment.

### Clinical characteristics of the six sub-groups

There were 83 patients in the low PQ group. According to their records, three were poisoned via dermal contact from spraying PQ, and the others attempted suicide by ingesting PQ; most presented with damage to the oral mucosa. The highest PQ concentration in the low-PQ group was 883 ng/mL, and six of the 83 patients died. The survival rate did not differ between the three treatment sub-groups. One patient, whose plasma PQ concentration at admission was 450 ng/mL, arrived at the hospital 19 h after ingestion of PQ, consequently suffering serious liver and kidney damage 3 days after admission. Since it was deemed that the patient would not improve, the patient’s family elected to discontinue treatment. The baseline parameters of patients in the low-PQ group indicated acute renal and liver damage, seen from a reduced urine volume, and elevated ALT and AST levels.

In patients with a plasma PQ concentration between 1,000 and 5,000 ng/mL (high-PQ group), the survival rate declined sharply to 51.85%. The survival rates in three sub-groups (HP, CRRT, and HP+CRRT) were 40.7%, 52%, and 62.1%, respectively (Table 1). A 34-year-old female patient with a baseline PQ concentration of 4,195 ng/mL arrived at the hospital 2 h after ingesting PQ, and HP treatment was initiated within 3.5 h. The patient’s PQ concentration rapidly decreased to 50 ng/mL after HP treatment. As the plasma PQ was rapidly cleared from the patient’s blood, kidney, liver, and lung damage was not extensive. After 26 days of treatment, the patient’s condition improved and she was discharged.

### Prognosis analysis

**Fig 1:**
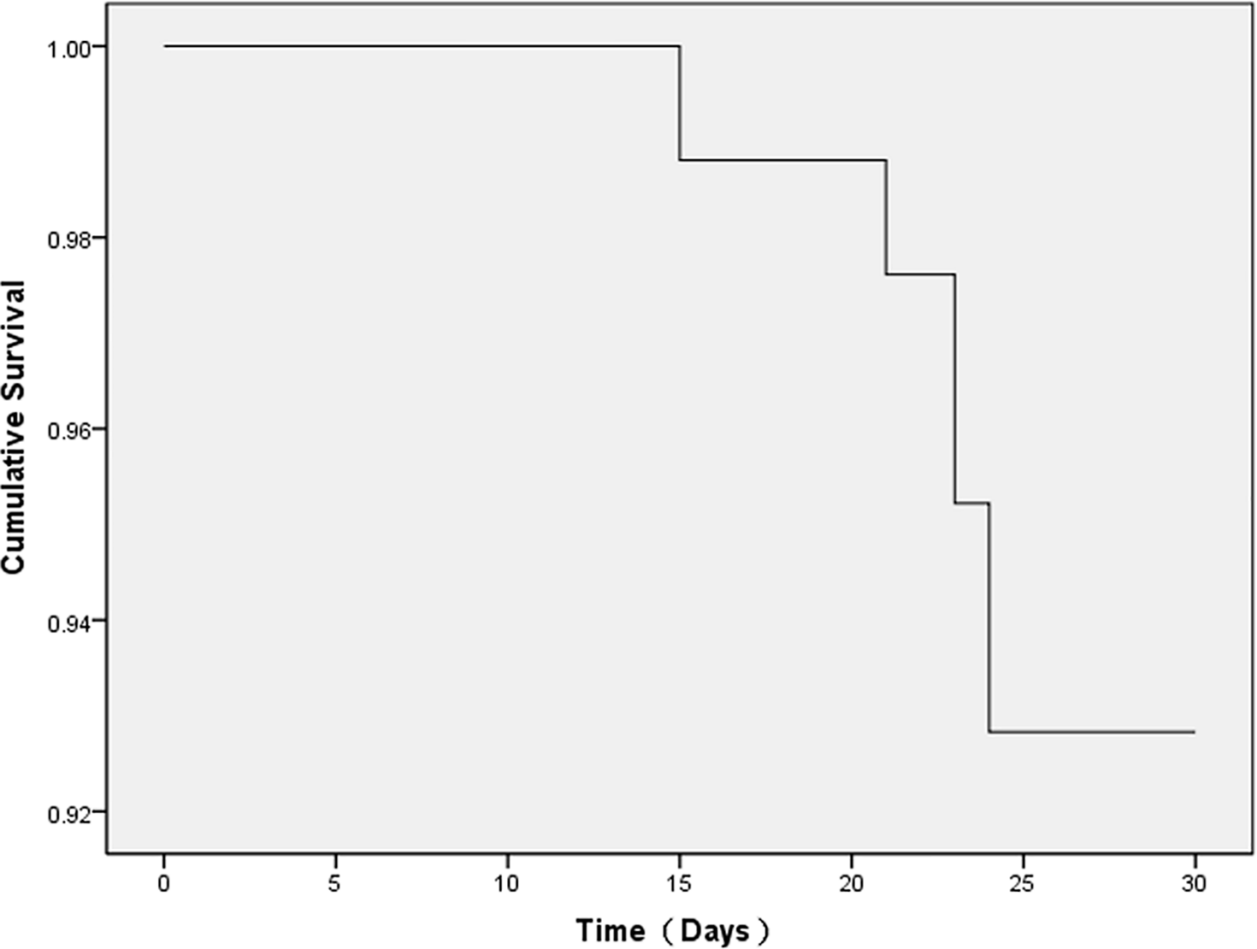
Survival time of 83 paraquat-poisoned patients (plasma paraquat levels: 50–1,000 ng/mL).

**Fig 2:**
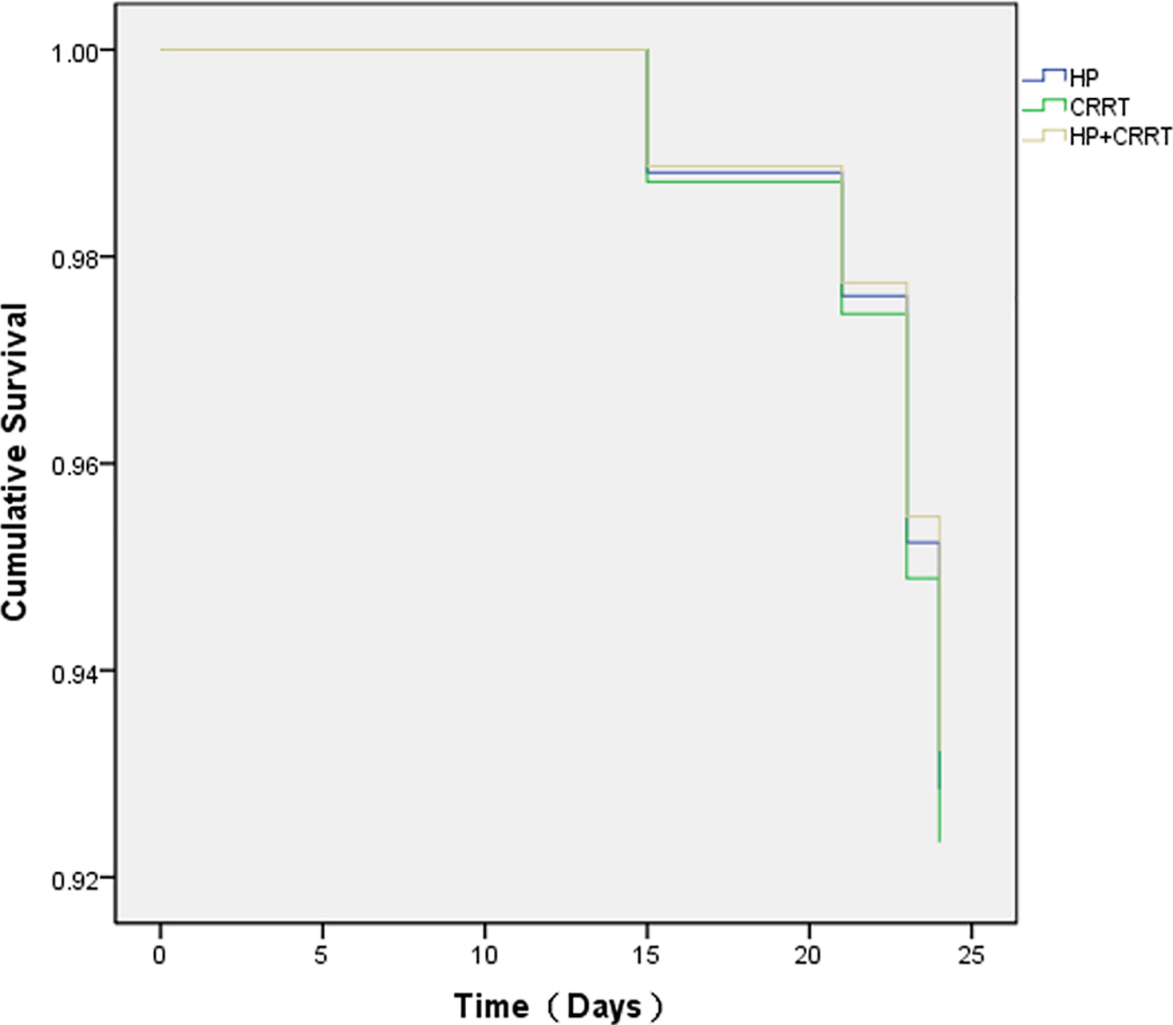
Comparison of survival curves for three treatments. Hemoperfusion (HP), continuous renal replacement therapy (CRRT), and combined treatment (HP+CRRT) in patients with plasma paraquat levels between 50 and 1,000 ng/mL. The chi-squared value between HP and CRRT was 1.056; *p*>0.05. The chi-squared value between HP and HP+CRRT was 1.136; *p*>0.05.

The life table of the 83 patients whose plasma PQ concentration ranged from 50 to 1,000 ng/mL is shown in Table 4. No patients died within 10 days of admission. Between 10 and 30 days post-admission, mortality rates were 7.1% in the HP sub-group, 7.7% in the CRRT sub-group, and 6.9% in the HP+CRRT sub-group. The survival time of patients in this group is shown in Fig 1. There were no significant differences in overall survival rates and in biomedical parameters during the first 10 days of hospitalization between the three treatment sub-groups, with the exception of total bilirubin levels. The Cox proportional hazards model for survival analysis found no significant differences in survival rates between the three treatment sub-groups (Fig 2), and showed that the three treatment methods were not related to prognosis (*p*>0.05). This indicates that at plasma PQ concentrations ranging from 50 to 1,000 ng/mL, the treatment protocol was not a sensitive index for patient prognosis.

**Table 4:** Fatality times and rates of paraquat-poisoned patients with low plasma paraquat levels (50–1,000 ng/mL, n = 83).

Treatment group	Death (<10 d)		Death (10–30 d)		Total Death	
	N	Fatality (%)	N	Fatality (%)	N	Fatality (%)
**HP**	0	0	2	7.1	2	7.1
**CRRT**	0	0	2	7.7	2	7.7
**HP+CRRT**	0	0	2	6.9	2	6.9

Abbreviations: HP, hemoperfusion; CRRT, continuous renal replacement therapy; HP+CRRT, combined treatment.

CRRT, continuous renal replacement therapy; HP+CRRT, combined treatment.

**Fig 3:**
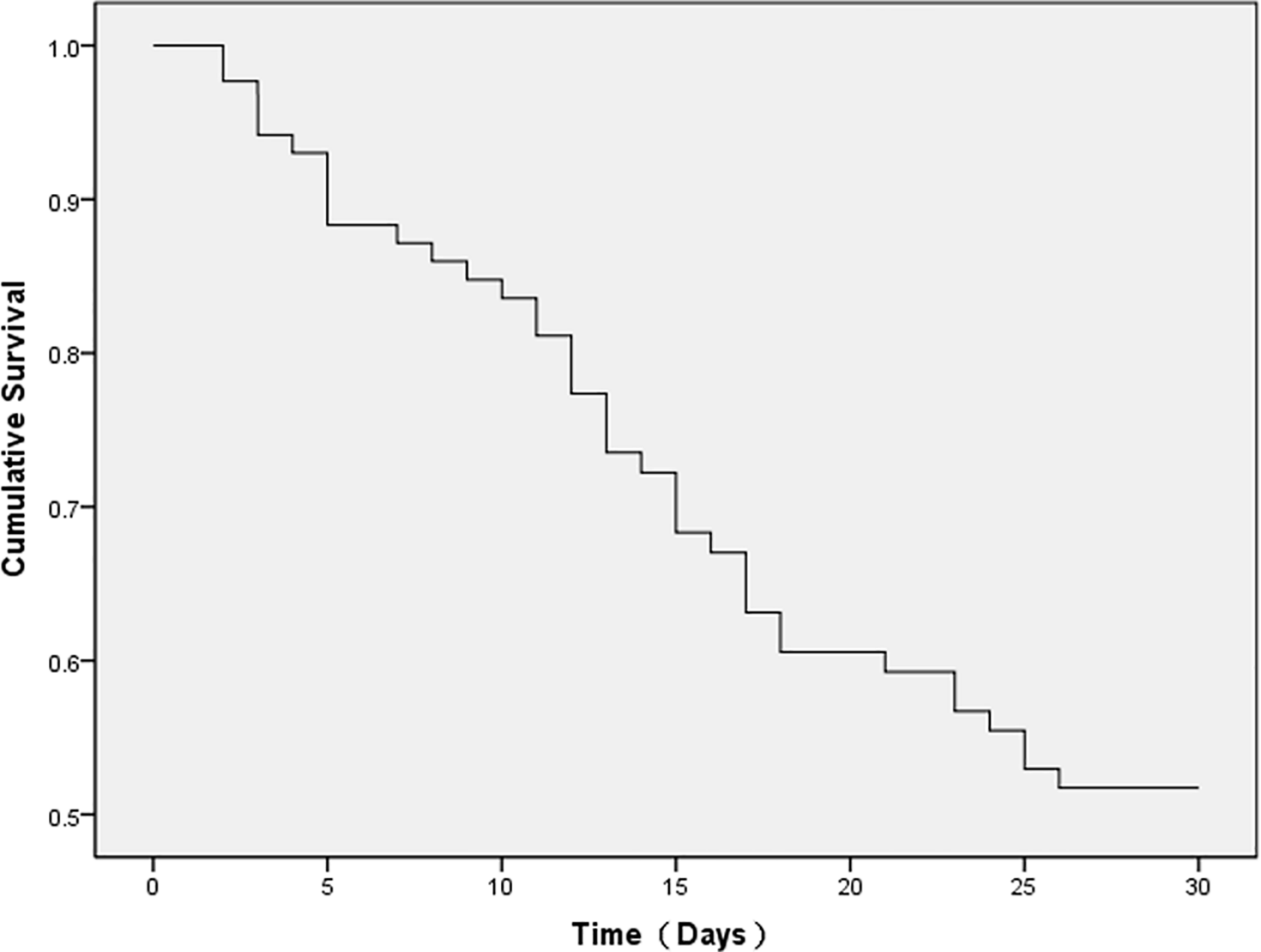
Survival time of 81 paraquat-poisoned patients (plasma paraquat levels: 1,000–5,000 ng/mL).

**Fig 4:**
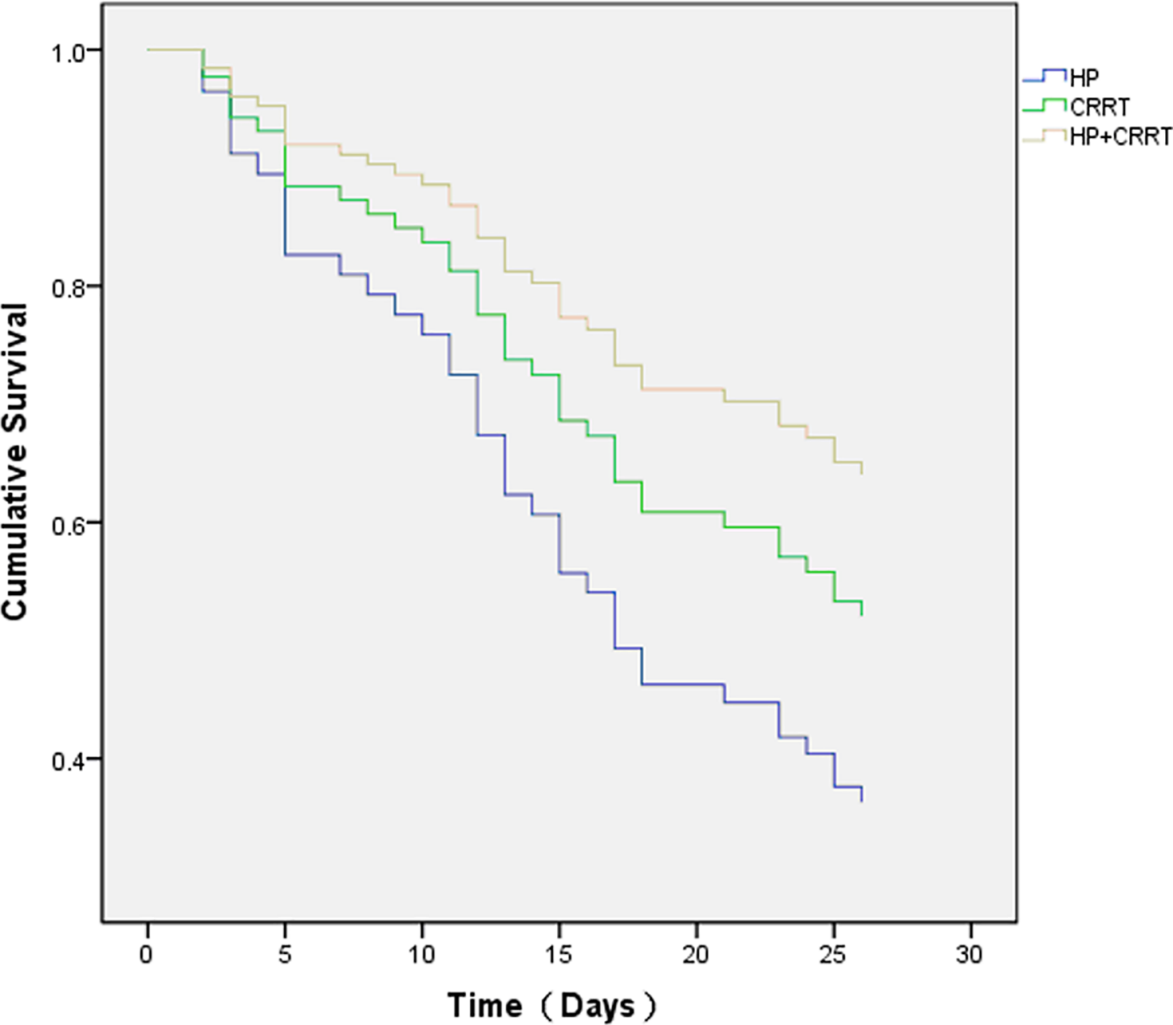
Comparison of survival curves for three treatments. Hemoperfusion (HP), continuous renal replacement therapy (CRRT), and combined treatment (HP+CRRT) in patients with plasma paraquat levels between 1,000 and 5,000 ng/mL. The chi-squared value between HP and CRRT was 1.466; *p*>0.05. The chi-squared value between HP and HP+CRRT was 2.276; *p*<0.05.

Eighty-one patients with high plasma PQ concentrations (1,000–5,000 ng/mL) were treated similarly. Within the first 10 days of hospitalization, the treatment sub-groups’ fatality rates were 25.9% in the HP sub-group, 16% in the CRRT sub-group, and 6.9% in the HP+CRRT sub-group (*p*<0.05); but the fatality rates on days 10–30 were higher, but did not differ between treatment sub-groups (*p*>0.05) (Table 5). Fig 3 shows the survival curve of patients in the high-PQ group. The Cox proportional hazards model for survival analysis found significant differences in survival rates between the three treatment sub-groups (Fig 4) and treatment methods were related to prognosis (*p*<0.05). This indicates that at plasma PQ concentrations ranging from 1,000 to 5,000 ng/mL, the treatment protocol is a sensitive index for patient prognosis; the combined HP+CRRT treatment yielded the highest survival rates.

**Table 5:** Fatality times and rates of paraquat-poisoned patients with high plasma paraquat levels (1,000–5,000 ng/mL, n = 81).

Treatment group	Death (<10 d)		Death (10–30 d)		Total Death	
	N	Fatality (%)	N	Fatality (%)	N	Fatality (%)
**HP**	7	25.9	9	33.3	16	59.2
**CRRT**	4	16	8	32	12	48
**HP+CRRT**	2	6.9	9	31	11	37.9

Abbreviations: HP, hemoperfusion

## Discussion

In high-PQ patients, the fatality rate within 10 days of hospital admission was lowest in the HP+CRRT group, and the CRRT sub-group’s mortality was lower than that of the HP sub-group. However, for patients with longer hospital stays (10–30 days), there was no significant difference in mortality between the three treatment sub-groups. In patients with low plasma PQ concentrations (50–1,000 ng/mL), there were no significant differences in survival rates between the three therapy sub-groups. These findings provide useful information for clinical application of HP and CRRT in patients with PQ intoxication.

PQ remains a popular pesticide in China, and there is no good antidote for acute PQ poisoning [1, 5]. During the first few hours of PQ intoxication, PQ cation radicals (PQ^+^ or PQ^++^) with a high affinity for alveoles directly damage the lungs and frequently cause death from respiratory failure [18, 19]. Roebuck [20] and Hensley et al. [21] reported that reactive oxygen species generated with acute PQ intoxication mediate cell signal transduction and cause inflammation and inflammatory cell infiltration, leading to liver and kidney damage. Other studies reported that PQ damaged mitochondria, released free iron from ferritin, and elevated production of reactive oxygen species, resulting in damage to multiple organs [22, 23]. However, the mechanism of PQ intoxication is not well-understood. Therapies usually include clearing plasma PQ and anti-oxidation and anti-inflammatory measures, but there is still no effective way to treat patients with acute PQ poisoning, and it is critical to develop new clinical approaches to further improve patient outcomes.

We previously analyzed plasma PQ concentrations in patients with PQ intoxication, and found that the initial PQ concentration was related to liver, kidney, and coagulation functions, and could be used as an index for assessing PQ prognosis [7]. Four PQ concentration ranges were evaluated, and PQ concentrations lower than 50 ng/mL were deemed not life-threatening, while concentrations exceeding 5,000 ng/mL were considered lethal, even if patients received early HP and comprehensive treatments. Therefore, in this study, we selected two ranges of plasma PQ concentrations (50–1,000 ng/mL and 1,000–5,000 ng/mL) to compare HP and CRRT. HP is currently considered the only effective approach for treating PQ intoxication, and has been broadly applied in clinical settings [16, 24, 25]. As in HP, CRRT achieves movement of solutes across a semi-permeable membrane [26, 27]. Due to CRRT’s slow diffusion, its convection overcomes the reduced removal rate of larger solutes seen in HP. To compare the efficacy of HP and CRRT, we set up three treatment sub-groups at the two ranges of plasma PQ levels, and further divided the data into three time points for comparing blood biochemical parameters and mortality rates. In the low-PQ group, some blood biochemical parameters varied over time in different treatment groups, but fatality rates did not. In contrast, in the high-PQ group, most liver and kidney indices improved significantly, and fatality rates during the first 10 days of hospitalization differed between treatment sub-groups.

Our study has some limitations. The number of cases was small, and the study population comprised only Asians. Owing to PQ’s rapid redistribution from the circulation to other compartments, the initial PQ determination may not have been reliable, and may have resulted in incorrect sub-grouping [28]. Plasma and urine concentrations have been reported to be effective parameters in predicting clinical outcome following acute PQ poisoning [29]. Therefore, measuring urine PQ levels may be required for better assessment in cases of PQ poisoning.

## Conclusions

The lack of a specific antidote for PQ poisoning makes improving the current treatment methods highly desirable. We found that CRRT therapy improved the prognosis of PQ-poisoned patients to a greater extent than HP when patients’ plasma PQ levels ranged from 1,000 to 5,000 ng/mL. Combining both treatments may increase the survival of PQ-poisoned patients, and should be considered a beneficial protocol in current clinical treatments.

## Supporting information

S1 File STROBE_checklist_v4_combined_PlosMedicine. (DOCX)

S2 File STROBE statement list answers. (DOC)

## References

[1] Hart TB. Paraquat—a review of safety in agricultural and horticultural use. Hum Toxicol. 1987; 6: 13–18. 3546082 10.1177/096032718700600103

[2] Senarathna L, Eddleston M, Wilks MF, Woollen BH, Tomenson JA, Roberts DM, et al Prediction of outcome after paraquat poisoning by measurement of the plasma paraquat concentration. QJM. 2009; 102: 251–259. doi: 10.1093/qjmed/hcp006. Epub 2009 Feb 19. 19228776 10.1093/qjmed/hcp006 PMC2659600

[3] Hong SY, Hwang KY, Lee EY, Eun SW, Cho SR, Han CS, et al Effect of vitamin C on plasma total antioxidant status in patient with paraquat intoxication. Toxicol Lett. 2002; 126(1): 51–59. 11738270 10.1016/s0378-4274(01)00431-3

[4] Myung W, Lee GH, Won HH, Fava M, Mischoulon D, Nyer M, et al Paraquat prohibition and change in the suicide rate and methods in South Korea. PloS One. 2015; 10, e0128980 doi: 10.1371/journal.pone.0128980. eCollection 2015. 26035175 10.1371/journal.pone.0128980 PMC4452788

[5] Sittipunt C. Paraquat poisoning. Respir Care. 2005; 50: 383–385. 15779152

[6] Rio MJ, Velez-Pardo C. Paraquat induces apoptosis in human lymphocytes: Protective and rescue effects of glucose, cannabinoids and insulin-like growth factor-1. Growth Factors. 2008; 26(1): 49–60. doi: 10.1080/08977190801984205 18365879 10.1080/08977190801984205

[7] Hong G, Hu L, Tang Y, Zhang T, Kang S, Zhao G, et al Prognosis and survival analysis of paraquat poisoned patients based on improved HPLC-UV method. J Pharmacol Toxicol Methods. 2016; 80: 75–81. doi: 10.1016/j.vascn.2016.05.010. Epub 2016 May 20. 27216136 10.1016/j.vascn.2016.05.010

[8] Suh GJ, Lee CC, Jo IJ, Shin SD, Lee JC, Min BG, et al Hemoperfusion using dual pulsatile pump in paraquat poisoning. Am J Emerg Med. 2008; 26(6): 641–648. doi: 10.1016/j.ajem.2007.09.022 18606314 10.1016/j.ajem.2007.09.022

[9] Lin JL, Lin-Tan DT, Chen KH, Huang WH, Hsu CW, Hsu HH, et al Improved survival in severe paraquat poisoning with repeated pulse therapy of cyclophosphamide and steroids. Intensive Care Med. 2011; 37(4): 1006–1013. doi: 10.1007/s00134-010-2127-7. Epub 2011 Feb 15. 21327593 10.1007/s00134-010-2127-7

[10] Amirshahrokhi K. Anti-inflammatory effect of thalidomide in paraquat-induced pulmonary injury in mice. Int Immunopharmacol. 2013; 17(2): 210–215. doi: 10.1016/j.intimp.2013.06.005. Epub 2013 Jun 26. 23810410 10.1016/j.intimp.2013.06.005

[11] Hu L, Hong G, Ma J, Wang X, Lin G, Zhang X, et al Clearance rate and BP-ANN model in paraquat poisoned patients treated with hemoperfusion. Biomed Res Int. 2015; 298253. doi: 10.1155/2015/298253. Epub 2015 Jan 28. 25695058 10.1155/2015/298253 PMC4324821

[12] Sun L, Li GQ, Yan PB, Liu Y, Li GF, Wei LQ. Prediction of outcome following paraquat poisoning by arterial lactate concentration-time data. Exp Ther Med. 2014; 8(2): 652–656. Epub 2014 Jun 11. doi: 10.3892/etm.2014.1773 25009635 10.3892/etm.2014.1773 PMC4079403

[13] Amano I, Inagaki Y. Complications and side effects of hemoperfusion. 1991; 49 Suppl: 649–654. 1808331

[14] Wu Y, Wang L, Meng L, Cao GK, Zhang Y. Evaluation of CRRT effects on pyemic secondary AKI by serum cartilage glycoprotein 39 and Annexin A1. Exp Ther Med. 2016; 12(5): 2997–3001. Epub 2016 Sep 9. doi: 10.3892/etm.2016.3691 27882106 10.3892/etm.2016.3691 PMC5103737

[15] Rico MP, Fernández Sarmiento J, Rojas Velasquez AM, Gonzalez-Chaparro LS, Gastelbondo Amaya R, Mulett Hoyos H, et al Regional citrate anticoagulation for continuous renal replacement therapy in children. Pediatr Nephrol. 2017; 32(4):703–711. doi: 10.1007/s00467-016-3544-9. Epub 2016 Nov 28. 27896442 10.1007/s00467-016-3544-9

[16] Shi X, Zhang Y, Wang Y. Impact of Xuebijing and ulinastatin as assistance for hemoperfusion in treating acute paraquat poisoning. Int J Clin Exp Med. 2015; 8(8): 14018–14023. eCollection 2015. 26550361 PMC4613046

[17] Chen GM, Chen YH, Zhang W, Yu Y, Chen JH, Chen J. Therapy of Severe Heatshock in Combination With Multiple Organ Dysfunction With Continuous Renal Replacement Therapy. Medicine (Baltimore). 2015; 94(31): e1212 doi: 10.1097/MD.0000000000001212 26252279 10.1097/MD.0000000000001212 PMC4616599

[18] Matthew H, Logan A, Woodruff MF, Heard B. Paraquat poisoning–lung transplantation. Br Med J. 1968; 3 (5621): 759–763. 4877735 10.1136/bmj.3.5621.759 PMC1989570

[19] Darke PG, Gibbs C, Kelly DF, Morgan DG, Pearson H, Weaver BM. Acute respiratory distress in the dog associated with paraquat poisoning. Vet Rec. 1977; 100(14): 275–277. 860382 10.1136/vr.100.14.275

[20] Roebuck KA. Oxidant stress regulation of IL-8 and ICAM-1 gene expression: differential activation and binding of the transcription factors AP-1 and NF-kappa B (Review). Int J Mol Med. 1999; 4(3): 223–230. 10425270 10.3892/ijmm.4.3.223

[21] Hensley K, Robinson KA, Gabbita SP, Salsman S, Floyd RA. Reactive oxygen species, cell signaling, and cell injury. Free Radic Biol Med. 2000; 28(10): 1456–1462. 10927169 10.1016/s0891-5849(00)00252-5

[22] Koskenkorva-Frank TS, Weiss G, Koppenol WH, Burckhardt S. The complex interplay of iron metabolism, reactive oxygen species, and reactive nitrogen species: insights into the potential of various iron therapies to induce oxidative and nitrosative stress. Free Radic Biol Med. 2013; 65: 1174–1194. doi: 10.1016/j.freeradbiomed.2013.09.001. Epub 2013 Sep 12. 24036104 10.1016/j.freeradbiomed.2013.09.001

[23] Jomova K, Valko M. Advances in metal-induced oxidative stress and human disease. Toxicology, 2011; 283(2–3): 65–87. doi: 10.1016/j.tox.2011.03.001. Epub 2011 Mar 23. 21414382 10.1016/j.tox.2011.03.001

[24] Xu XL, Wang W, Song ZJ, Ding H, Duan XH, Meng HC, et al Imaging in detecting sites of pulmonary fibrosis induced by paraquat. World J Emerg Med. 2011; 2(1): 45–49. 25214982 PMC4129732

[25] Liu Y, Qiu Q, Ge Y. Effects of hemoperfusion on plasma concentration and histopathological changes in paraquat poisoning rabbits. Zhonghua Lao Dong Wei Sheng Zhi Ye Bing Za Zhi. 2011; 29(10): 735–739. 22357487

[26] Villa G, Di Maggio P, De Gaudio AR, Novelli A, Antoniotti R, Fiaccadori E, et al Effects of continuous renal replacement therapy on linezolid pharmacokinetic/pharmacodynamics: a systematic review. Crit Care. 2016; 20(1): 374 doi: 10.1186/s13054-016-1551-7 27863531 10.1186/s13054-016-1551-7 PMC5116218

[27] Kirwan CJ, Hutchison R, Ghabina S, Schwarze S, Beane A, Ramsay S, et al Implementation of a Simplified Regional Citrate Anticoagulation Protocol for Post-Dilution Continuous Hemofiltration Using a Bicarbonate Buffered, Calcium Containing Replacement Solution. Blood Purif. 2016; 42(4): 349–355. Epub 2016 Nov 19. doi: 10.1159/000452755 27866200 10.1159/000452755

[28] Baud FJ, Houze P, Bismuth C, Scherrmann JM, Jaeger A, Keyes C. Toxicokinetics of paraquat through the heart-lung block. Six cases of acute human poisoning. J Toxicol Clin Toxicol. 1988; 26 (1–2): 35–50. 3385847 10.3109/15563658808995396

[29] Seok S, Kim YH, Gil HW, Song HY, Hong SY. The time between paraquat ingestion and a negative dithionite urine test in an independent risk factor for death and organ failure in acute paraquat intoxication. J Korean Med Sci. 2012; 27(9): 993–998. doi: 10.3346/jkms.2012.27.9.993. Epub 2012 Aug 22. 22969243 10.3346/jkms.2012.27.9.993 PMC3429840

