# Supplementary material for: Effects of hemoperfusion and continuous renal replacement therapy on patient survival following paraquat poisoning
Source: PLoS One. 2017 Jul 13;12(7):e0181207. doi: 10.1371/journal.pone.0181207 (PMC5509301; doi:10.1371/journal.pone.0181207)
Supplement: S2 File — (DOC) [file pone.0181207.s002.doc]

STROBE Statement List Answer

**Title and abstract**

**Item No.1** (a) and (b)

(a)Answer: The commonly used terms in the title or the abstract were found in **Page No.** P1, P2. **Relevant text from manuscript**: “hemoperfusion (HP); continuous renal replacement therapy (CRRT); paraquat (PQ).”

(b)Answer: The informative and balanced summary in the title or the abstract were found in **Page No.** P2, P3. **Relevant text from manuscript**: “This study assessed the HP and CRRT on the survival of PQ-poisoned patients with plasma PQ levels below 5,000 ng/mL. In the early stages of PQ poisoning, CRRT is effective in reducing patient fatality rates, particularly when combined with HP.”

**Introduction**

Background/rationale **Item No.2**

Answer: We have explained the scientific background and rationale for the investigation in **Page No.** P3, P4. **Relevant text from manuscript**: “PQ intoxication is a serious public health problem, and results in an estimated 60–70% mortality [3, 4]. Because no specific antidote or conclusively effective treatment having been found, an acute ingestion of 7–8 mL PQ can cause serious effects such as liver, lung, kidney, and heart failure, leading directly to death [5, 6]. Various therapeutic techniques are applied to clinical intervention in PQ poisoning. HP has been validated as the most effective method to clear PQ from blood [11, 12]. However, HP may result in complications such as thrombocytopenia, leukopenia, hypoglycemia, and some reduction in clotting factors. Risk of bleeding is higher because of high heparin doses and a reduction in platelets and clotting factors [13]. With slow blood flow rates and access through a central venous catheter placed in one of the large central veins, CRRT can keep blood from clotting even if heparin and regional citrate are used for anti-coagulation [14, 15].”

Objectives **Item No.3**

Answer: The study’s objective was discovered in **Page No.** P4. **Relevant text from manuscript**: “Although HP has been validated as an effective treatment, the effects of combined treatment with HP and CRRT on the survival of patients with PQ poisoning at different concentrations, particularly PQ levels from 1,000 to 5,000 ng/mL, is still unclear. Based on long-time monitoring of the effects of HP and CRRT at different PQ concentrations, we performed a retrospective study on the prognosis and survival of PQ-poisoned patients admitted to the First Afﬁliated Hospital of Wenzhou Medical University in China. The various clinical indices included the time of treatment, and biochemistry parameters were analyzed to assess the effects of HP and CRRT on survival time.”

**Methods**

Study design **Item No.4**

Answer: We thought the key elements of study design early in the paper that both inclusion criteria and exclusion of the study were drawn up on the basis of patient initial plasma PQ concentrations. Another was how to ensure the randomness of the grouping objects. **Relevant text from manuscript’s Page No.** P5 and P6: During this period, 215 patients with PQ poisoning were admitted to the hospital; 164 patients were eventually included in the study on the basis of inclusion and exclusion criteria.” “All data obtained from the PQ-poisoned patients were recorded and standardized in a Microsoft Excel spreadsheet by two emergency department physicians who were unaware of the purpose of this investigation.”

Setting **Item No.5**

Answer: The study’s recruits with PQ poisoning were from Department of Emergency, the First Afﬁliated Hospital of Wenzhou Medical University in China. Patient data were recorded for thirty days including periods of recruitment, exposure and follow-up. Inpatient data from the medical records, and follow-up patients were regularly tested once a week and recorded results. **Relevant text from manuscript’s Page No.** P5 and P6: “We reviewed the medical records of patients admitted to the First Afﬁliated Hospital of Wenzhou Medical University in China with acute PQ poisoning between January 1, 2011 and May 31, 2015.” “All data obtained from the PQ-poisoned patients were recorded and standardized in a Microsoft Excel spreadsheet by two emergency department physicians who were unaware of the purpose of this investigation.”

Participants **Item No.6**

(*a*)Answer: The study belongs to *Cohort study*. The eligibility criteria were listed in **Page No.** P5. The participants were PQ-poisoning patients from Department of Emergency, through initial plasma PQ concentrations detection and the poisoning history of inquiry. After the patient is discharged, we tested the patient once a week and recorded results. For very patient, the observation period was 30 days. **Relevant text from manuscript’s Page No.** P5 and P6: “Inclusion criteria were (1) a clear history of PQ poisoning and PQ detected in blood; (2) patient age between 15 and 65 years; (3) hospital admission within 24 h of poisoning without pretreatment; (4) no history of serious chronic disease; (5) no contraindications of HP treatment. Exclusion criteria were (1) PQ poisoning patients who died within 24 h; (2) patients with other pesticide poisoning; (3) pregnant patients; (4) patients with a history of alcoholism; and (5) patients with serum PQ levels above 5000 ng/mL.” “The PQ-poisoned patients were divided into two different groups (low PQ [LPQ] group and high PQ [HPQ] group) according to their initial plasma PQ concentrations. PQ concentrations in the LPQ group were below 1000 ng/mL. Each group was randomly sub-divided into three sub-groups based on treatment: HP sub-group, CRRT sub-group, and HP+CRRT sub-group.”

(*b*)Answer: The participants were from same *Cohort* accordance to Inclusion criteria (see above), and randomly sub-divided into every group. **Relevant text from manuscript’s Page No.** P5 and P6: Besides the (*a*)Answer above, “The size of each group was as follows: (1) LPQ-HP, 28 cases; (2) LPQ-CRRT, 26 cases; (3) LPQ-HP-CRRT, 29 cases; (4) HPQ-HP, 27 cases; (5) HPQ-CRRT, 25 cases; (6) HPQ-HP-CRRT, 29 cases.”

Variables **Item No.7**

Answer: The blood biochemistry indicated vital signs of patients. Through initial plasma PQ concentrations detection and the poisoning history of inquiry, we diagnosed PQ-poisoning patients. The survival time was the key variable for comparing the two treatments (HP and CRRT). The treatment outcomes’ variable was defined as categorical variables (1: Better; 0: Worse or death). Other variables were made separately for age, sex, time from PQ ingestion to arrival at the hospital, time from PQ ingestion to ﬁrst gastric lavage, time from PQ ingestion to ﬁrst HP/CRRT/HP+CRRT treatment, and liver, kidney, and coagulation function. **Relevant text from manuscript’s Page No.** P9: “Categorical variables were analyzed using the chi-squared test. The variables were analyzed via nonparametric test and the other indices were analyzed via ANOVA. To assess the relationship between treatment protocols and mortality, the multivariate Cox regression survival curves with the Cox proportional hazard model were generated. The primary dependent variable was the survival time, measured in days. The treatment outcomes of PQ-poisoned patients were entered as categorical variables (1: Better; 0: Worse or death). Multivariate adjustment was made separately for age, sex, time from PQ ingestion to arrival at the hospital, time from PQ ingestion to ﬁrst gastric lavage, time from PQ ingestion to ﬁrst HP/CRRT/HP+CRRT treatment, and liver, kidney, and coagulation function.”

Data sources/ measurement **Item No.8**

Answer: For all variables, we firstly defined the variables and inputted the variables’ data in SPSS. Secondly, Categorical variables (age, sex etc) were analyzed using the chi-squared test. After looking for the Descriptive Statistics in Analyze, we selected the chi-squared test in General linear model for reviewing different sub-groups. The blood biochemistry indices were analyzed via ANOVA. We selected the ANOVA in Comparison mean for reviewing different sub-groups. The Fatality times and rates’ variables were analyzed using the multivariate Cox regression survival curves with the Cox proportional hazard model. We selected the Cox regression in Generating function for reviewing different sub-groups. Thirdly, checking analyses results, the level of signiﬁcance was set at *p*<0.05. **Relevant text from manuscript’s Page No.** P9: “The data were analyzed using SPSS (version 19.0, IBM SPSS, Armonk, NY, USA). Results are presented as means ±standard deviation. The level of signiﬁcance was set at *p*<0.05. Categorical variables were analyzed using the chi-squared test. The variables were analyzed via nonparametric test and the other indices were analyzed via ANOVA. To assess the relationship between treatment protocols and mortality, the multivariate Cox regression survival curves with the Cox proportional hazard model were generated. The primary dependent variable was the survival time, measured in days. The treatment outcomes of PQ-poisoned patients were entered as categorical variables (1: Better; 0: Worse or death). Multivariate adjustment was made separately for age, sex, time from PQ ingestion to arrival at the hospital, time from PQ ingestion to ﬁrst gastric lavage, time from PQ ingestion to ﬁrst HP/CRRT/HP+CRRT treatment, and liver, kidney, and coagulation function.”

Bias **Item No.9**

Answer: The recording data’s physicians don’t know the purpose of this investigation, and randomly sub-grouping. **Relevant text from manuscript’s Page No.** P6: “Each group was randomly sub-divided into three sub-groups based on treatment:” “All data obtained from the PQ-poisoned patients were recorded and standardized in a Microsoft Excel spreadsheet by two emergency department physicians who were unaware of the purpose of this investigation.”

Study size **Item No.10**

Answer: Our statistics were 5 years in the emergency department and the result of the joint efforts of many people. **Relevant text from manuscript’s Page No.** P5: “We reviewed the medical records of patients admitted to the First Afﬁliated Hospital of Wenzhou Medical University in China with acute PQ poisoning between January 1, 2011 and May 31, 2015. During this period, 215 patients with PQ poisoning were admitted to the hospital; 164 patients were eventually included in the study on the basis of inclusion and exclusion criteria.”

Quantitative variables **Item No.11**

Answer: The quantitative variables firstly were defined and inputted their data in SPSS software. Then we selected the ANOVA in Comparison mean for reviewing different sub-groups. The groupings were random for reducing human efforts. **Relevant text from manuscript’s Page No.** P6: “Each group was randomly sub-divided into three sub-groups based on treatment:”

Statistical methods **Item No.12**

(a)Answer: Categorical variables were analyzed using the chi-squared test. Blood biochemistry indices were analyzed via ANOVA. The multivariate Cox regression survival curves were used in the analyses of Fatality times and rates’ variables. All data could be saw on **Item No.8.** **Relevant text from manuscript’s Page No.** P9: “The data were analyzed using SPSS (version 19.0, IBM SPSS, Armonk, NY, USA). Results are presented as means ±standard deviation. The level of signiﬁcance was set at *p*<0.05. Categorical variables were analyzed using the chi-squared test. The variables were analyzed via nonparametric test and the other indices were analyzed via ANOVA. To assess the relationship between treatment protocols and mortality, the multivariate Cox regression survival curves with the Cox proportional hazard model were generated. The primary dependent variable was the survival time, measured in days. The treatment outcomes of PQ-poisoned patients were entered as categorical variables (1: Better; 0: Worse or death). Multivariate adjustment was made separately for age, sex, time from PQ ingestion to arrival at the hospital, time from PQ ingestion to ﬁrst gastric lavage, time from PQ ingestion to ﬁrst HP/CRRT/HP+CRRT treatment, and liver, kidney, and coagulation function.”

(b)Answer: For sub-groups, we compared the corresponding subgroups by statistical methods.

(c)Answer: Missing data were directly set as the delete label at setting variables in SPSS software.

(d)Answer: For the Cohort study, the loss to follow-up could be set as the delete label at setting variables in SPSS software.

(e)Answer: The reliability of the test result was set to 95%. That was that the level of signiﬁcance was set at *p*<0.05.

**Results**

Participants **Item No.13**

(a)Answer: According to the selected standard, 164 of the 215 PQ-poisoning patients met the criteria and sub-grouped. For follow-up patients discharged within 30 days, we have tested the follow-up patients once a week and recorded results.

(b)Answer: basing on Exclusion criteria, a total of 51 PQ patients were excluded from the study. **Relevant text from manuscript’s Page No.** P5: “During this period, 215 patients with PQ poisoning were admitted to the hospital; 164 patients were eventually included in the study on the basis of inclusion and exclusion criteria.”

(c)Answer: The flow diagram of participants below.


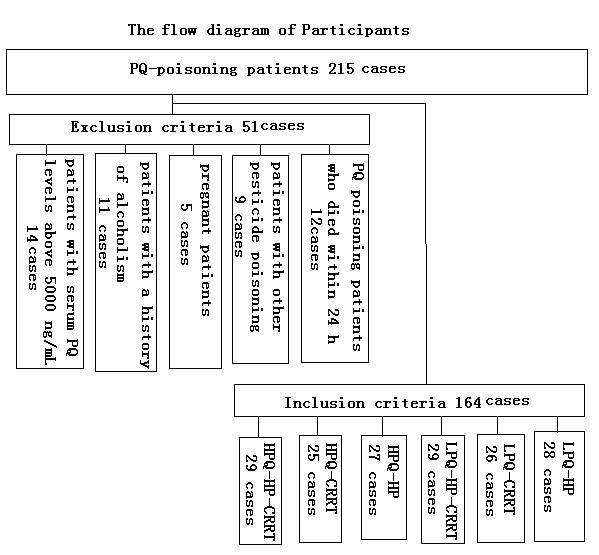


Participants **Item No.14**

(a)Answer: 197 of 215 PQ-poisoning participants live in Wenzhou, Zhejiang province in China, other migrant workers. Before PQ poisoning, the participants were in good physical condition including farmers (85%), civil servants (3%), students (1%) and other workers (11%).

(b)Answer: the two participants’ data missed because of unable to contact the two follow-up patients, which one was sub-grouped into LPQ-HP, another in HPQ-HP-CRRT.

(c)Answer: The follow-up participants’ time was average sixteen days in Cohort study. The143 of 164 PQ-poisoning patients underwent hospitalization and follow-up period in thirty days, and 19 patients hospitalized for 30 days.

Participants **Item No.15**

Answer: At low plasma paraquat levels (50–1,000 ng/mL, n=83), there were not detat for the PQ-poisoning patients in ten days. But between 10 and 30 days, each sub-group (HP; CRRT; HP+CRRT) had a total of two deaths, respectively. **Relevant text from manuscript’s Page No.** P19: **Table 4.** At high plasma paraquat levels (1000–5,000 ng/mL, n=81), the number of deaths in each sub-group (HP; CRRT; HP+CRRT) within 10 days was 7, 4 and 2, respectively, and during 10 to 30 days, every sub-group deaths’ number being 9, 8 and 9 PQ-poisoning patients. **Relevant text from manuscript’s Page No.** P20: **Table 5.**

Participants **Item No.16**

(a)Answer: For fatality times and rates, blood biochemistry indices and clinical characteristics of PQ-poisoning patients, these data were unadjusted to evaluate the effect of different treatment methods. There were many factors affecting clinical investigation, and its was difficult to control. In order to provide no difference between the characteristics of each sub-group (95% confidence interval), we have set up the Exclusion criteria and analyzed the general clinical data of each group. These data would help us to correctly analyze different therapies. **Relevant text from manuscript’s Page No.** P10, P14: **Table 1. “**There was no statistically significant difference (*p*>0.05) in sex, age, admission time, time to first gavage, time to first HP/CRRT/HP+CRRT treatment, length of hospital stay, survival, or initial or improved biochemical parameters between the groups. The 81 patients with plasma PQ levels of 1000–5000 ng/mL exhibited comparable biochemical parameters, with the exception of survival or improvement, as calculated by variance analysis. These data indicate that the treatments were comparable across the different sub-groups.**” “**Table 2 shows the biochemical parameters of patients with plasma PQ concentrations ranging from 50 to 1,000ng/mL. At admission (baseline), these parameters did not differ among the three treatment sub-groups (HP, CRRT, and HP+CRRT).**” “**In patients with plasma PQ levels ranging from 1,000 to 5,000ng/mL, we analyzed the effect of different treatments (HP, CRRT, and HP+CRRT) at three time points (baseline, day 5, and day 10). As shown in Table 3,**”**

(b)Answer: When continuous variables were categorized, category boundaries of different groups had partial overlap, but the continuous variables were presented as means ± standard deviation, which had no effect for analyzing these variable. **Relevant text from manuscript’s Page No.** P10-P14: **Table 1, Table 2, Table 3.**

(c)Answer: The estimate of relative risk was used to a meaningful time period.

Participants **Item No.17**

Answer: The difference between the two sub-groups (HP, CRRT, and HP+CRRT) was compared with the corresponding statistical methods. Its’ confidence interval was set 95%.

**Discussion**

Key results **Item No.18**

Answer: In the early stages of PQ poisoning, CRRT is effective in reducing patient fatality rates, particularly when combined with HP. Our data could be useful in increasing survival in acute PQ poisoning patients. **Relevant text from manuscript’s Page No.** P21: “In high-PQ patients, the fatality rate within 10 days of hospital admission was lowest in the HP+CRRT group, and the CRRT sub-group’s mortality was lower than that of the HP sub-group. However, for patients with longer hospital stays (10–30 days), there was no significant difference in mortality between the three treatment sub-groups. In patients with low plasma PQ concentrations (50–1,000 ng/mL), there were no signiﬁcant differences in survival rates between the three therapy sub-groups. These findings provide useful information for clinical application of HP and CRRT in patients with PQ intoxication.”

Participants **Item No.19**

Answer: There were many limiting factors in our study. Such as the number of cases was small, and the study population comprised only Asians. The more reliable conclusions were based a lot of cases and human species in the study. Another, non random grouping method might result in potential bias or imprecision for the study’s conclusion. **Relevant text from manuscript’s Page No.** P22: “Our study has some limitations. The number of cases was small, and the study population comprised only Asians. Owing to PQ’s rapid redistribution from the circulation to other compartments, the initial PQ determination may not have been reliable, and may have resulted in incorrect sub-grouping [28]. Plasma and urine concentrations have been reported to be effective parameters in predicting clinical outcome following acute PQ poisoning [29]. Therefore, measuring urine PQ levels may be required for better assessment in cases of PQ poisoning.”

Participants **Item No.20**

Answer: Although there were many limitations in our study, we have adopted a variety of scientific methods to reduce the potential impact for the conclusion, such as Inclusion criteria, Exclusion criteria, Random grouping etc. Statistical analysis concluded that there was no difference between subgroups in the study. The literature showed that research has been done on HP and CRRT applications. We have investigated the effect of initial plasma concentrations of PQ on survival, and the paper was a relevant research that has been studied. **Relevant text from manuscript’s Page No.** P22, P23: “Our study has some limitations. The number of cases was small, and the study population comprised only Asians. Owing to PQ’s rapid redistribution from the circulation to other compartments, the initial PQ determination may not have been reliable, and may have resulted in incorrect sub-grouping [28]. Plasma and urine concentrations have been reported to be effective parameters in predicting clinical outcome following acute PQ poisoning [29]. Therefore, measuring urine PQ levels may be required for better assessment in cases of PQ poisoning.”

Participants **Item No.21**

Answer: HP and CRRT for PQ poisoning patients have been used in clinic. However, the role of HP and CRRT in patients with different initial concentrations of PQ has not been reported. We found that CRRT therapy improved the prognosis of PQ-poisoned patients to a greater extent than HP when patients’ plasma PQ levels ranged from 1,000 to 5,000ng/mL. Combining both treatments may increase the survival of PQ-poisoned patients, and should be considered a beneficial protocol in current clinical treatments. **Relevant text from manuscript’s Page No.** P23: “The lack of a specific antidote for PQ poisoning makes improving the current treatment methods highly desirable. We found that CRRT therapy improved the prognosis of PQ-poisoned patients to a greater extent than HP when patients’ plasma PQ levels ranged from 1,000 to 5,000ng/mL. Combining both treatments may increase the survival of PQ-poisoned patients, and should be considered a beneficial protocol in current clinical treatments.”

**Other information**

Funding **Item No.22**

Answer: This work was supported by fund of the key construction academic subject (Medical Innovation) of Zhejiang province (11-CX26), China. The present article is based the original study “Prognosis and survival analysis of paraquat poisoned patients based on improved HPLC-UV method. J Pharmacol Toxicol Methods. 2016; 80: 75-81.” **Relevant text from manuscript’s Page No.** P24: **References** 7.
